# Pangenome evaluation of gene essentiality in *Streptococcus pyogenes*

**DOI:** 10.1128/spectrum.03240-23

**Published:** 2024-07-16

**Authors:** Magnus G. Jespersen, Andrew J. Hayes, Steven Y. C. Tong, Mark R. Davies

**Affiliations:** 1 Department of Microbiology and Immunology, The University of Melbourne, at the Peter Doherty Institute for Infection and Immunity, Melbourne, Australia; 2 Department of Infectious Diseases, The University of Melbourne, at the Peter Doherty Institute for Infection and Immunity, Melbourne, Australia; 3 Victorian Infectious Diseases Service, The Royal Melbourne Hospital, at the Peter Doherty Institute for Infection and Immunity, Melbourne, Australia; Politecnico di Torino, Torino, Piemonte, Italy

**Keywords:** genomics, *Streptococcus pyogenes*, gene disruption, pangenome

## Abstract

Bacterial species often consist of strains with variable gene content, collectively referred to as the pangenome. Variations in the genetic makeup of strains can alter bacterial physiology and fitness. To define biologically relevant genes of a genome, genome-wide transposon mutant libraries have been used to identify genes essential for survival or virulence in a given strain. Such phenotypic studies have been conducted in four different genotypes of the human pathogen *Streptococcus pyogenes*, yet challenges exist in comparing results across studies conducted in different genetic backgrounds and conditions. To advance genotype to phenotype inferences across different *S. pyogenes* strains, we built a pangenome database of 249 *S*. *pyogenes* reference genomes. We systematically re-analyzed publicly available transposon sequencing datasets from *S. pyogenes* using a transposon sequencing-specific analysis pipeline, Transit. Across four genetic backgrounds and nine phenotypic conditions, 355 genes were essential for survival, corresponding to ~24% of the core genome. Clusters of Orthologous Genes (COG) categories related to coenzyme and lipid transport and growth functions were overrepresented as essential. Finally, essential operons across *S. pyogenes* genotypes were defined, with an increased number of essential operons detected under *in vivo* conditions. This study provides an extendible database to which new studies can be added, and a searchable html-based resource to direct future investigations into *S. pyogenes* biology.

**IMPORTANCE:**

*Streptococcus pyogenes* is a human-adapted pathogen occupying restricted ecological niches. Understanding the essentiality of genes across different strains and experimental conditions is important to direct research questions and efforts to prevent the large burden of disease caused by *S. pyogenes*. To this end we systematically reanalyzed transposon sequencing studies in *S. pyogenes* using transposon sequencing-specific methods, integrating them into an extendible meta-analysis framework. This provides a repository of gene essentiality in *S. pyogenes* which was used to highlight specific genes of interest and for the community to guide future phenotypic studies.

## INTRODUCTION

*Streptococcus pyogenes* is a human-adapted pathogen estimated to cause more than 517,000 deaths and more than 720 million cases of superficial infections such as pyoderma or pharyngitis each year (1). The development of vaccines to prevent *S. pyogenes* is an area of active research, with no product yet commercialized, despite clear health and economic incentives (2–6). As a result of the high burden of disease, research to understand the fundamental biology of this pathogen is required to define which gene(s) are central to the survival of the pathogen irrespective of the ecological niche occupied or clinical presentation associated with *S. pyogenes*. This knowledge can be used to identify new drug targets or better understand the vast amounts of data generated using high-throughput methods, such as transcriptomics and other omics techniques.

Transposon sequencing is one high-throughput method that has been successfully used to probe gene essentiality in bacteria. While multiple versions of this method have been developed (7–12), common is the use of modified transposable elements (transposons), which are inserted semi-randomly into the genome. If a transposon inserts in or close to a gene, this is likely to cause a gene knock-out. Through this approach, a mutant library is generated with each bacterial cell having a transposon inserted “randomly” in its genome, which can then be screened using high-throughput sequencing methods for regions of increased or decreased transposon insertion rate. The rate of transposon insertion indicates the essentiality of a specific sequence under a given condition and is detected by increased or decreased mutant survival rates (13). In *S. pyogenes,* transposon sequencing has been used to examine gene essentiality under different *in vivo* and *in vitro* conditions (14–22). These studies have highlighted important genes related to survival, colonization, and infection in varying environments and conditions including human challenge and murine skin or soft tissue infections (18, 23).

One of the limitations of transposon sequencing has been an inability to systematically compare studies where strain type, conditions, and informatic processing are not uniform. An additional complexity is the fact that *S. pyogenes* is a genetically diverse organism and total gene content varies across different strains (2). Recent transposon studies have focused on producing collections of transposon mutant libraries and analyzed these to elucidate pangenome essentiality dynamics, where interactions with or between accessory genes can lead to the essentiality of one or more genes in certain genetic backgrounds (24–26). However, defining gene essentiality across different genetic backgrounds requires adaptive evolutionary frameworks to drive future research efforts. In *S. pyogenes* few transposon sequencing studies have been put into a co-analysis to increase understanding across data sets. When studies are compared, results often originate from different analysis pipelines possibly introducing additional biases. Here we conducted a systematic re-analysis of transposon sequencing studies from *S. pyogenes*, with methods specifically developed for transposon sequencing (27, 28), and subsequent weighting of each gene in terms of its essentiality. These findings serve as an extendible database for gene essentiality, provided in an html-based searchable format, to guide future research into defining the biology of *S. pyogenes* infections.

## RESULTS

A total of nine transposon sequencing studies have been conducted in *S. pyogenes* (14–22). Of these, five studies representing nine data sets were analyzed in this study after applying the following exclusion criteria: microarray-based (14); raw reads were not publicly available (17, 21); condition-restricted preventing comparability (20, 22); and low saturation of transposon insertion (15). These data sets included represented four *S*. *pyogenes* strains of three *emm* types: two *emm*1 strains (5448 and MGAS2221), one *emm* 28 (MGAS27961), and one *emm*49 (NZ131). The data sets covered *in vitro* (*n* = 4) and *in vivo* (*n* = 4) growth conditions with *emm*1 and *emm*28 tested across both (Fig. S1). The *emm*49 strain was only tested *in vitro*. Finally, one dataset of *emm1* consisted of an *in vitro* Zn^2+^ challenge, which was not included in *in vitro* essentiality calculations due to the added stressor. *In vitro* and *in vivo* conditions covered multiple different media types and sites of infection in different model organisms (Table 1). To unify separate data sets we applied optimal trimming, alignment, and count of insertions with the tool Transit (27, 28) taking Transposon type into account (Table S1). This method allowed for systematic assessment of gene essentiality across experimental discrepancies (refer to Materials and Methods). Differences in essentiality calls (essential, conditionally essential, and non-essential) across data sets and different transposon types were compared to assess potential variation induced by transposon types (Fig. S2).

**TABLE 1:** Transposon sequencing studies of *S. pyogenes*^*a*^

Study (PMID)	Strain	*emm* (MLST)	Condition	Infection model	*In vivo*/ *in vitro*	Transposon type	Included
25996237	5448	1 (28)	THY	–	*In vitro*	Himar1-like	Yes
25996237	NZ131	49 (1073)	THY	–	*In vitro*	Himar1-like	Yes
25996237	Alab49	53 (11)	THY	–	*In vitro*	Himar1-like	No, low saturation
28832676	5448	1 (28)	Skin and soft tissue	Mouse	*In vivo*	Himar1-like	Yes
29104937	MGAS2221	1 (28)	Human saliva	–	*In vitro*	TN5-like	No, no reads available
30321240	5448	1 (28)	THY + hGIIA	–	*In vitro*	Himar1-like	No, condition restricted
30667377	MGAS27961	28 (52)	CDM	–	*In vitro*	Tn5-like	Yes
30667377	MGAS2221	1 (28)	CDM	–	*In vitro*	Tn5-like	Yes
30667377	MGAS27961	28 (52)	Necrotizing myositis	Non-human primate	*In vivo*	Tn5-like	Yes
30667377	MGAS2221	1 (28)	Necrotizing myositis	Non-human primate	*In vivo*	Tn5-like	Yes
30936502	5448	1 (28)	RPMI + Zn^2+^	–	*In vitro*	Himar1-like	Yes
30936502	5448	1 (28)	THY + hGIIA	–	*In vitro*	Himar1-like	No
32200972	MGAS27961	28 (52)	Vaginal colonization	Non-human primate	*In vivo*	Tn5-like	Yes
32200972	MGAS27961	28 (52)	Uterine wall infection model	–	*In vitro*	Tn5-like	No, condition restricted
36374114	MGAS2221	1 (28)	THY + penicillin G	–	*In vitro*	TN5-like	No, no reads available
36374114	MGAS2221	1 (28)	THY + ceftriaxone	–	*In vitro*	TN5-like	No, no reads available

^*a*^ General overview of studies utilizing transposon sequencing to examine *S. pyogenes*. hGIIA, human group IIA secreted phospholipase A2; THY, Todd-Hewitt medium supplemented with yeast extract; CDM, chemically defined medium. Exclusion criteria: microarray-based; no publicly available raw reads; condition-restricted preventing comparability; and low saturation of transposon insertion. Dash (-) indicates information not relevant.

### *S. pyogenes* reference pangenome

**Fig 1:**
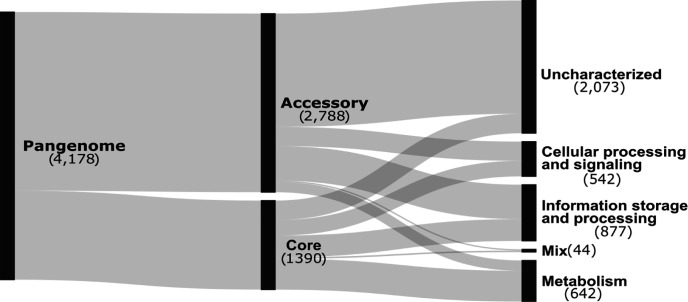
Pangenome and functional classification across an *S. pyogenes* reference pangenome. Sankey flow diagram from left to right illustrating how genes from the pangenome of 249 complete genomes, are distributed among core and accessory categories, and different functional categories based on COG categories.

As existing transposon sequencing studies were performed in different genetic backgrounds, a pangenome built from all publicly available complete reference genomes allows relationships of coding regions to be assigned across different studies. The rationale for incorporating all available reference genomes was to increase the broader utility of the data set by extrapolating gene essentiality across reference genomes and additional strains of *S. pyogenes* and to have a framework to observe potential lineage adaptations to variable genes across the population (accessory genes). The *S. pyogenes* “reference” pangenome was constructed from 249 publicly available reference genomes (Supplementary file 1) using Panaroo (29), and excluding potential plasmids. The genomes represent 103 different *emm* types. Functional annotation of the pangenome was obtained using the eggNOG mapper and the eggNOG database (30, 31). Of the 4,178 genes identified in the “reference” pangenome, 1,390 (1,390/4,178%–33.3%) genes were identified as core genes, encoded in ≥99% of isolates (≥246 genomes) (Fig. 1). The fraction of core genes is similar to previous pangenome analyses of *S. pyogenes* (2, 32, 33). A total of 2,788 pangenome genes were found in <99% of isolates and are variable in presence across genomes, therefore these are termed accessory genes (Fig. 1). The four strains used for transposon sequencing studies encode a subset of genes from the pangenome (*n* = 1,999). The genes from the four strains represent a coverage of 47.8% (1,999/4,178) of the “reference” pangenome (core and accessory) (Data S1; HTML S1). Annotations from eggNOG assigned Clusters of Orthologous Genes (COG) categories which at times were collated into major categories based on related functions, to simplify interpretation (Table S2). Both core and accessory genes were predicted to encode products in each of the major COG categories (Fig. 1).

### Importance of the core genome and unveiled strain adaptations

**Fig 2:**
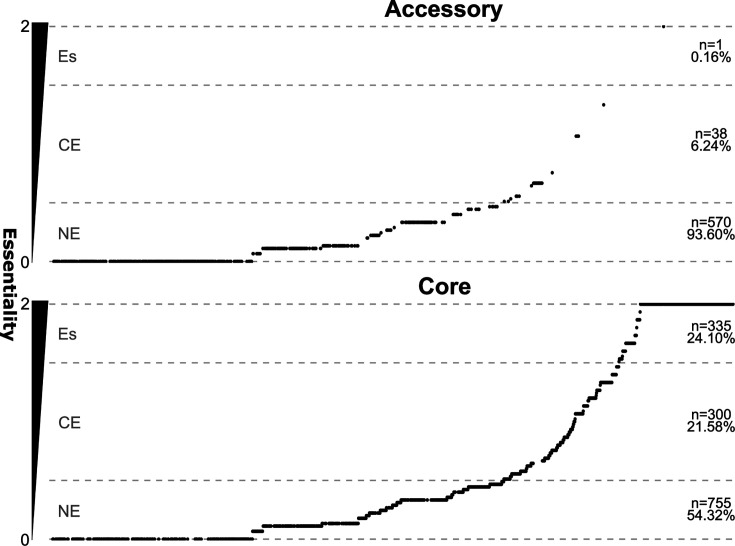
Gene essentiality in a subset of pangenome genes represented by experimentally tested strains. Distributions of gene essentiality scores for core and accessory genes encoded in at least one of the four experimentally tested strains. Genes are ordered horizontally by increasing the essentiality score from left to right. Vertically, the essentiality score increases from the bottom to the top and horizontal gray dashed lines indicate borders between quantitative categories of essentiality [non-essential (NE) (0 ≤ essentiality score≤ 0.5), conditionally-essential (CE) (0.5 < essentiality score< 1.5), essential (Es) (1.5 ≤ essentiality score≤ 2)], as well as the minimum (essentiality score = 0) and maximum (essentiality score = 2) essentiality values indicated by the bottom and top lines, respectively.

To identify essential genes across the *S. pyogenes* pangenome, a gene essentiality score was determined. This score is a semi-quantitative measure of essentiality across data sets calculated for a specific gene or operon. Essentiality scores are defined as non-essential (value “0”); conditionally-essential (value “1”) or essential (value “2”). Numerical values are used to enable statistical calculations to be defined at two major levels: (i) within data sets of the same lineage/*emm* type and (ii) between data sets derived from different lineage/*emm* types: specifically, the mean essentiality score was calculated from essentiality calls within nine publicly available data sets; *emm*1 (*n* = 4), *emm*28 (*n* = 4), and *emm*49 (*n* = 1). For each gene, the mean of all essentiality scores derived from each *emm* type (*emm*1, *emm*28, *emm*49) were then used to derive the lineage (*emm*) specific essentiality score (Data S2; HTML S2). As the essentiality score is the mean of means, the lineage (*emm*) specific essentiality score is represented in decimal notation across the data sets analyzed. This method aimed to weigh each *emm* lineage equally to avoid bias from sampling. For the 1,999 genes represented at least once across the four strains used in the nine transposon mutagenesis studies, core genes had a significantly larger essentiality score compared to accessory genes (Mann-Whitney U test: *P* < 2.2 × 10^−16^) (Fig. 2). The median essentiality score for core genes was 0.44 (Q1 = 0.11; Q3 = 1.4) meaning that a little over half of the core genome is seemingly not essential (essentiality score ≤ 0.5, *n* = 755, 755/1,390, 54%), with around a quarter being essential (essentiality score ≥ 1.5, *n* = 335, 335/1,390, 24%). For accessory genes, the median was 0.00 (Q1 = 0.00; Q3 = 0.133).

### Enriched functional categories and poorly characterized genes

**Fig 3:**
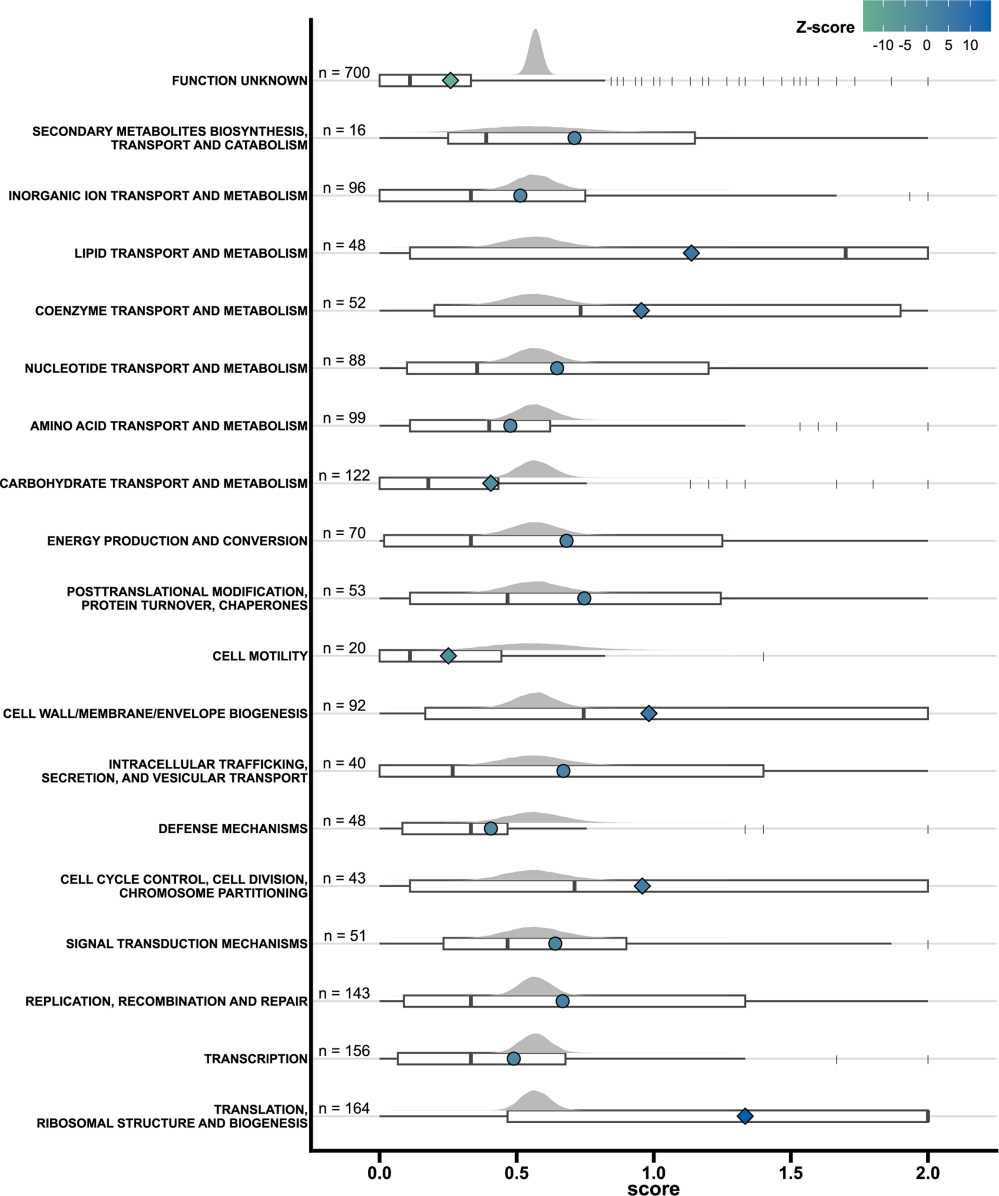
Essentiality enrichment of COG categories. Distributions of essentiality (horizontal axis) for each represented COG category (vertical axis). Gray distributions are formed by 50,000 mean values calculated on random resamplings of essentiality scores. Boxplots plots beneath every gray distribution represent the observed distribution of essentiality scores for a given category, with outliers given as vertical lines. Each point (circle and diamond) represents the mean value for the observed essentiality scores for each COG category. The mean essentiality score point is illustrated as a diamond if the mean has a Z-score >2 of <−2 relative to the resampled distribution. The color of mean points is relative to their Z-score, which is given by the legend. The number of genes contributing to each category is given next to the vertical axis.

Dividing genes by their COG categories reveals a difference in essentiality among functional categories (Fig. 3). Several COG categories have essentiality scores that differ from the expected, compared to a random sampling. Categories with lower-than-expected essentiality cover genes with unknown function and carbohydrate metabolism. The finding for carbohydrate metabolism is supported by previous observations from metabolic modeling, where the versatility of carbohydrate utilization was noted (34). Categories more essential than expected concern translation machinery, cell cycle-related categories, and coenzyme and lipid metabolism. An example from one of these categories is genes encoding functions in the fatty acid biosynthesis (*fab*) genes. These 13 genes, organized into three operons (o_0583, o_0584, and o_0585 in S119) (35, 36), are essential across all conditions. The *fab* genes have previously been linked to virulence (37, 38). The results presented here and in previous studies stress *fabT* and the remaining *fab* genes as potential targets for narrow-range therapeutics (35), highlighting how increased knowledge about cross-lineage fundamental functions of *S. pyogenes* can direct discovery and research into therapeutic targets.

Although the COG categories can point to general functions of increased importance, almost all categories have at least one gene essential across all tested conditions. This is also true for the genes assigned to the unknown function category. Although assigned to this category, some of these genes have been studied in other streptococcal species. Genes with poor annotation and large essentiality scores in this data set can guide efforts to study genes essential for *S. pyogenes* biology. An example of such a gene is *glpF2* (also called *Aagp*) (SF370 tag: SPY_RS07685/Spy1854, 5448 tag: SP5448_RS01610) with an essentiality of 1.6 (*emm*1: 1.8, *emm*28: 2, *emm*49: 1). *glpF2* has been identified as an atypical aquaglyceroporin facilitating the transport of hydrogen peroxide and likely glycerol (39). In *Streptococcus suis glpF2* was identified as important during infection of mice using a knockout strain, and in pigs by transposon mutagenesis (39, 40). Additionally, a *glpF2* knockout displayed a decreased lag phase duration during growth at 42°C in a rich medium (40). *glpF2* is notably found as having a decreased transcript abundance level when comparing multiple M1_Global_ and M1_UK_ strains, prompting the need for a better understanding of *glpF2* and its potential involvement in virulence, survival, and competition in a population (41, 42).

For accessory genes, 55% (*n* = 336/609) were found to have an essentiality score of 0 (Q1 = 0.0; median = 0.0; Q3 = 0.13). Only a single accessory gene was found in the highest quarter of the essentiality score range, compared to 335 core genes. This is consistent with the evolutionary conservation of core genes in the *S. pyogenes* population and suggests that the loss of function of any of the 335 core genes is lethal for the organism. The single accessory gene found in the upper quarter of the essentiality score range was of particular interest as it is missing in some *S. pyogenes lineages* but is seemingly essential for growth in all strains and conditions tested in the transposon mutagenesis studies to date. The gene was annotated by eggNOG as *secretion and acid tolerance D* (*satD*) (SF370 tag: SPY_RS04905/SPy1171, 5448 tag: SP5448_RS04940). *satD* was found to be essential across *emm*1, *emm*28, and *emm*49 strains, but is an accessory gene across the population of complete *S. pyogenes* genomes (178/249, ~71%). In *Streptococcus mutans*, *satD* and remaining *sat* genes have been associated with acid stress tolerance (43, 44). However, *satD* and related genes have not been studied in other streptococci. The example of *satD* showcases the importance of transposon sequencing meta-analyses, and how the integration of a pangenomic framework can expose strain-dependant adaptations otherwise missed when examining only a single strain or lineage in isolation.

### Stricter essentiality requirements under *in vivo* conditions

**Fig 4:**
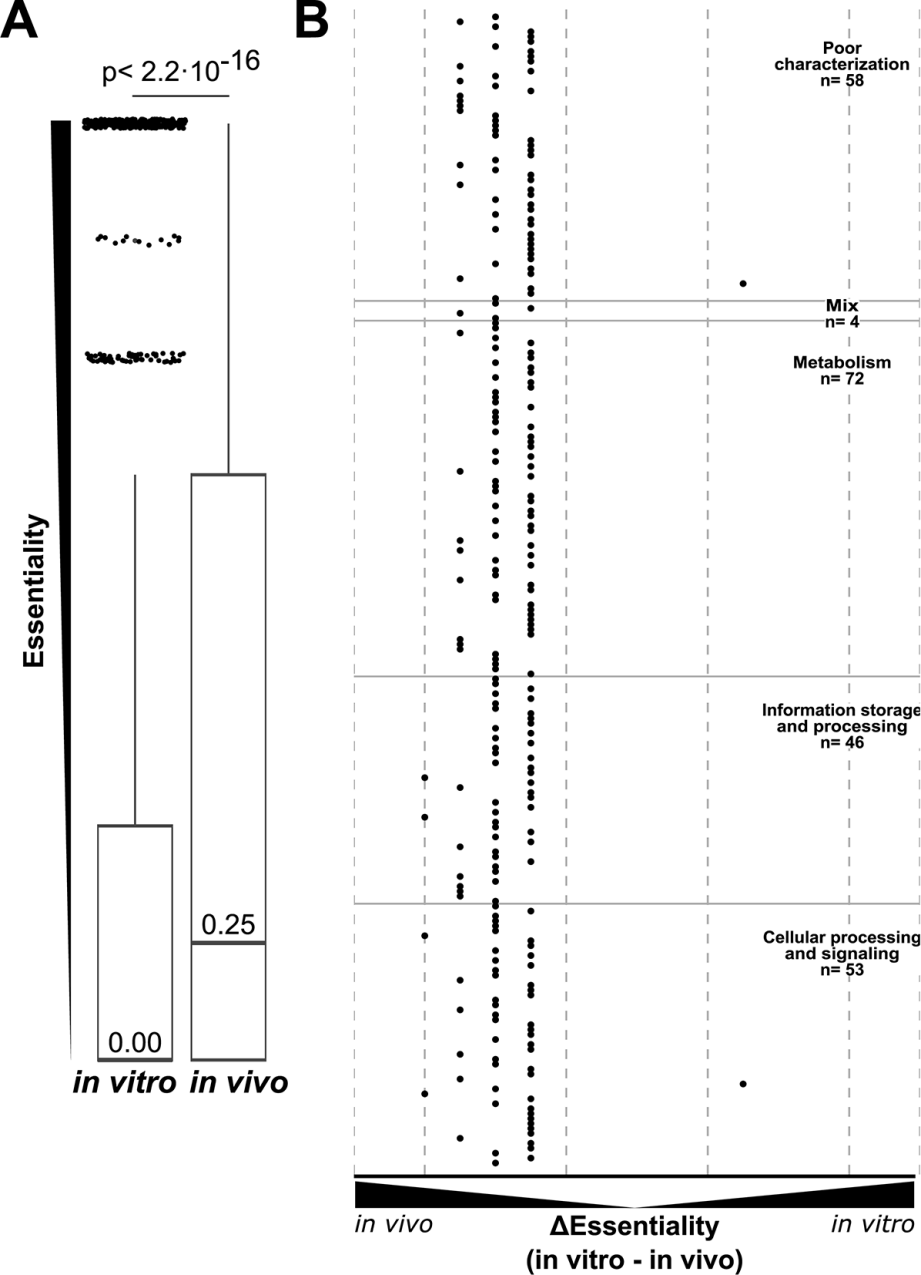
Differential gene essentiality across macro conditions: (**A**)Boxplot of gene essentiality scores based on *in vitro* and *in vivo* experimental conditions. Outliers (essentiality score≥ 1.5 inner quartile range) are marked by black points. The *P*-value for a Wilcoxon’s paired signed rank test between the two categories is given. (**B**)Distribution of genes with large differences in essentiality score (≥0.5) across *in vitro* and *in vivo* conditions (*n* = 245). Vertical dashed lines indicate differences of 2, 1.5, and 0.5, with the black dashed line indicating 0 difference between *in vivo* and *in vitro*. Major functional categories are given for points and separated by horizontal black lines. Points left of the black vertical dashed line have increased the essentiality score *in vivo*. Conversely, points right of the line have increased essentiality scores *in vitro*. In the lower-left corner adjusted *P*-values for pairwise chi-squared tests among well represent functional categories are given, initial chi-squared *P*-value = 8.4 × 10^−3^. Categories are shortened as follows: metabolism (Meta), information storage and processing, and cellular processing and signaling.

To assess gene essentiality between *in vivo* and *in vitro* conditions, we calculated the mean essentiality score within each condition not correcting for strain genotype. When comparing the distributions of essentiality of genes across *in vivo* (Q1 = 0.25; median = 0.25; Q3 = 1.25) and *in vitro* (Q1 = 0.00; median = 0.00; Q3 = 0.50) conditions, genes under *in vivo* conditions had a significantly increased essentiality score (paired Wilcoxon signed-rank test: *P* < 2.2 × 10^−16^) (Fig. 4A). These findings suggest that under *in vivo* conditions, *S. pyogenes* is more sensitive to gene knock-out and loss of function compared to *in vitro* conditions. Examining genes identified 233 (233/1,999, 11.6%) with large variation in essentiality score (>0.5) between *in vivo* and *in vitro* conditions (Fig. 4B). Only two genes were of increased essentiality under *in vitro* conditions. This was contrasted with 231 genes with greatly increased essentiality scores under *in vivo* conditions (Fig. 4B; Data S3). Using this metric we can hone into examples of poorly annotated core genes that may have important functions under *in vivo* conditions. One such example is a homolog to an RNA-binding protein CvfD from *S. pneumoniae* (45), which can be found in *S. pyogenes* (SF370 tag: SPY_RS06755/SPy1619, 5448 tag: SP5448_RS02795). This gene has an increased essentiality score under *in vivo* conditions compared to *in vitro* conditions. In *S. pneumoniae*, *cvfD* has been linked to virulence in a mouse model of invasive pneumonia and is found to regulate 144 mRNA transcripts. The *S. pyogenes* homolog of *cvfD* is uncharacterized but could be important in regulating expression at a post-transcriptional level under *in vivo* conditions (45).

### Contextualizing gene essentiality with operon architecture

**Fig 5:**
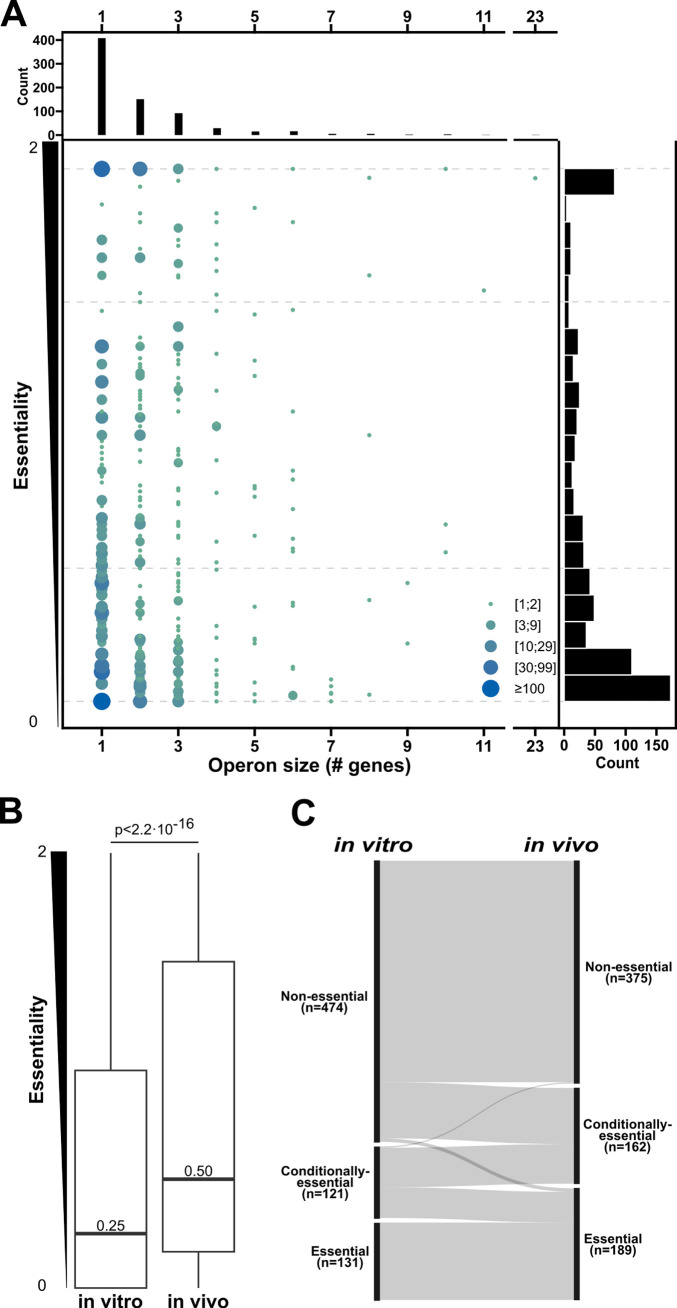
Operon essentiality for *S. pyogenes*: (**A**)distribution of size and essentiality score for operons (*n* = 732) from S119. The size and color of points correspond to a number of operons at a given position, relative to the legend. Distributions for each variable are given along the outer axis. (**B**)Boxplots for the distribution of essentiality score for operons under *in vitro* and *in vivo* conditions. Medians are given and the *P*-value for a paired Wilcoxon signed rank test is given. (**C**)Sankey flow diagram for change of qualitative categories of essentiality scores for operons under *in vitro* and *in vivo* conditions. Categories are based on essentiality in the following order: non-essential (essentiality score ≤ 0.5), conditionally essential (0.5 < essentiality score > 1.5), and essential (essentiality score ≥ 1.5). Gray links between the two columns (*in vitro* and *in vivo*) indicate the operons’ position in the given essentiality categories across conditions.

Many genes in bacteria are components of and are evolutionarily maintained within polycistronic operons. These genomic features can be highly important due to transcriptional suppression and promotion mechanics and genome compactness. To assess operons and their essentiality, we used operon structures as defined in *emm*1 strain S119 (36). Of the 750 operons identified in S119, 726 (96.8%) were successfully mapped to the pangenome of the 249 complete genomes. Mapped S119 operons encompassed 405 monocistronic and 321 polycistronic operons. The systematic comparison of pangenomes and gene essentiality enables an interrogation of the maintenance of essential genes in operon structures. The essentiality score of an operon was defined as the mean essentiality score for all genes in the operon (Data S4; HTML S3). Of monocistronic operons, 15% were essential (essentiality score > 1.5, *n* =61/405) with 17% of polycistronic operons deemed essential (essentiality score > 1.5, *n* = 56/321). Essential genes were found overrepresented in polycistronic operons compared to monocistronic operons (polycistronic 264/1,020 = 26%, monocistronic 71/407 = 17%, *P* = 0.0041), non-essential and conditionally-essential genes statistically were equally distributed. There was a wide range of operon essentiality scores across operons of differing sizes, with no clear correlation between operon and essentiality score (Fig. 5A). A linear regression model found the size of an operon to be a poor predictor of essentiality, R^2^ = 9.01 × 10^−3^ (*n* = 726). With multiple essential genes (*n* = 52) found in monocistronic operons and operon size found to be a weak predictor of operon essentiality, operon structure does not appear to be correlated with essentiality.

We next examined the influence of growth conditions, by calculating an *in vivo* and *in vitro* essentiality score for each operon (Data S4; HTML S3). Similar to findings for individual genes, comparing operon essentiality scores under different conditions showed the *in vivo* essentiality score (Q1 = 1.67; median = 0.50; Q3 = 1.50) to be significantly higher than the *in vitro* score (Q1 = 0.00; median = 0.25; Q3 = 1.00) (paired Wilcoxon signed-rank test: *P* < 2.2 × 10^−16^) (Fig. 5B).

Operons were next categorized into three qualitative groups based on essentiality score. Non-essential, operons with an essentiality score ≤ 0.5; conditionally-essential, operons essential in few strains and conditions (essentiality score >0.5 and <1.5) and; essential, operons with an essentiality score ≥ 1.5, indicating the operon to be essential in most strains and conditions. Division of operons into essentiality categories under *in vivo* and *in vitro* conditions separately demonstrated that operon essentiality can vary across conditions, with some operons being in different essentiality categories across *in vivo* and *in vitro* conditions (Fig. 5C). These operons and the genes within them may be of interest in understanding the physiology and survival of *S. pyogenes*. As an example, the operon for the Arginine deaminase pathway (o_0507 in S119), which has been found important during skin infection (46), has an increase in essentiality score of ≥0.5 for all genes *in vivo* compared to *in vitro*, with an increase in essentiality ~1 for the entire operon. Operons that have not been previously identified as important under *in vivo* conditions can also be discovered in the same way, potentially allowing new insights into *S. pyogenes* during infection conditions. One such is the S119 operon o_0575 encoding *pptA* and *pptB* (also called *ecsAB*, SF370 tag: SPY_RS07195/SPy1728, and SPY_RS07200/SPy1729, 5448 tags: SP5448_RS02105 and SP5448_RS02110). These two genes have been shown to export competence pheromones in *S. pyogenes* (47) but have not been linked to importance under *in vivo* conditions. *pptAB* are of low essentiality under *in vitro* conditions but increase to a moderate essentiality score under *in vivo* conditions. In *Streptococcus equi* subspecies *equi pptAB* is important for survival in horse blood cultures but not in hydrogen peroxide (48).

## DISCUSSION

One of the challenges of bacterial population biology is to identify important common functions among populations where gene content is variable, and evolution is ongoing. To facilitate broader analytical resources to the *S. pyogenes* research community, systematic analytical frameworks of functional genomic studies such as transposon mutagenesis studies, undertaken across different conditions and strain backgrounds, represent an important tool in defining gene essentiality in *S. pyogenes*. The fact that *S. pyogenes* is divided into hundreds of genomic lineages with varying and often poorly characterized gene content complicates the ability to define common genetic markers at a population level (2). This is emphasized by the varied hypotheses to explain the epidemiological differences between *S. pyogenes* lineages that have been puzzling researchers for decades (49, 50). However, with the sparse number of data sets (*n* = 9) and *emm* lineages (*n* = 3) represented in transposon sequencing studies, much is still to be learned about lineage-specific differences in essentiality of genes in the context of adaptation to different conditions or niches. In this work, only cross lineage *in vivo,* and *in vitro,* essentiality scores were calculated. To facilitate broader inferences from these three *emm* lineages to other lineages we constructed a pangenome using Panaroo, a recent pangenome tool that incorporates gene synteny into its analytical framework. The pangenome constructed from 249 complete reference genomes displayed a similar core genome size as previous analyses (2, 33), yet advances in these studies through the use of enhanced data sets and analytical approaches. With future studies, the inclusion of more strains and conditions that can be analyzed within the same pangenome framework will provide new opportunities to interpret essentiality across lineages. Additionally, essentiality scores within the same strain compared across *in vivo* and *in vitro* conditions could give a deeper understanding of how a strain adapts to these two different macro conditions.

To date, there has been no systematic re-analysis comparing the individual transposon sequencing data sets across *S. pyogenes*. As existing studies are constructed using different protocols it can be challenging to compare and integrate results across studies. Furthermore, little work has been done to investigate how batch effects may influence transposon sequencing, and how to control them. The Zero-Inflated Negative Binomial method from the Transit package, used to analyze Himar1-like transposon libraries, allows for co-variates to be included in the statistical model, but this requires nuanced experimental design and does not lend itself to this meta-analysis (28). Other biases known to affect transposon sequencing studies are the choice of transposons and the subsequent method of analyzing the differential presence of transposons across the genome (13, 28). How to best handle specific transposons, and which to choose is an ongoing field of research (13, 51). In this paper, we have chosen methods specifically developed to analyze transposon sequencing data, rather than the common but likely problematic approach of using RNA-seq analysis methods. The methods used here incorporate information on the transposon used in producing the data and hence reduce the bias that can be introduced from the method of analysis. The rationale for our study is to provide a framework for comparative analysis of transposon sequencing in the context of the exponential increase in *S. pyogenes* population genomics landscape (pangenomics). The identification of a single essential accessory gene, *satD*, highlights how the framework presented here, using a pangenome and transposon sequencing, allows for the investigation of possible lineage adaptations encoded by accessory genes. Furthermore, the integration of operon structures of *S. pyogenes* indicated different requirements between *in vivo* and *in vitro* conditions, with operons having a significantly greater essentiality score under *in vivo* conditions. While the number of publicly available data sets is currently low, we find additional differences and signatures within a pangenome landscape that were not a feature of the original study designs. The identification of lineage adaptation genes, important core genes, and new leads for increased understanding highlight the usefulness of transposon sequencing and pangenomics in combinations. With more research in transposon sequencing-specific methods and how to better compare data sets, such approaches could be highly valuable for research in a range of bacterial species.

Here we have undertaken a major reanalysis and assessed results from a repository of gene essentiality conducted across different *S. pyogenes emm* lineages and conditions. This has been integrated into a pangenome of complete genomes and operon structure to increase the usefulness of the data set. Additionally, the method used here to calculate essentiality can be extended to include new studies and update essentiality scores. Future studies will hopefully broaden our understanding of mechanisms underlying gene essentiality across additional strains and conditions, as has been done in other species (24–26).

## MATERIALS AND METHODS

### Pangenome construction and functional annotation

Two hundred and fifty-five complete genomes of *S. pyogenes* were collected from RefSeq as of 15 January 2021. Quality control of misassembles was done using Socru v.2.2.4 (52) and identifying spurious additional chromosomes (contigs) not related to extrachromosomal elements meant that the final number of reference genomes analyzed in this study was 249. A pangenome was constructed using Panaroo and flags: clean-mode set to strict, initial percent identity to 90% (−i 0.9), and maximum length difference to 95% (−l 0.95) (29). Functional annotations for each pangenome cluster identified by Panaroo were obtained by running eggNOG-mapper v.2.1.3 using the eggNOG database v5.0 (30, 31).

### Transposon sequencing analysis

Raw sequencing data were acquired from the sequence read archive (SRA) using the SRA Toolkit (github.com/ncbi/sra-tools). FastQC v.0.11.9 (www.bioinformatics.babraham.ac.uk/projects/fastqc/) and seqkit v.2.2.0 were used to assess the quality of raw fastq files (53). Depending on transposon type (Tn5 or Himar1) and study, data were trimmed to isolate genomic DNA from reads using Cutadapt v.3.8.6 (54) (https://journal.embnet.org/index.php/embnetjournal/article/view/200). Tn5-like transposons were trimmed using the sequence AGATCGGAAGAG, and flags: -e 2, --no-indels, -O 10, and --action trim. For Untrimmed Himar1 transposon reads the sequence: ACAGGTTGGATGATAAGTCCCCGGTCTGACACATCTCCCTAT was used with flag: -m 10. Subsequent quality filtering was conducted using seqkit seq -t dna -Q 34. Alignment and counting of reads were done using the Transit TPP tool v.3.2.6 modified to run bowtie v.1.3.1 as the alignment tool, using flags -S, -m 1, -k 1, -n 0 (27, 28, 55). For each lineage used in the original studies a corresponding RefSeq reference genome was used for alignment and construction of Prot_tables (Transit specific annotations format) related to the pangenome annotations (NZ131: GCF_000018125.1, Alab49: GCF_000230295.1, 5448: GCF_001021955.1, MGAS2221: GCF_012572265.1, MGAS27961: GCF_004010855.1). For all input conditions of Tn5 libraries essential genes were determined using the Tn5Gaps with -r means [Tn5 methods from Transit (56)]. For Tn5 data sets conditionally essential genes were identified based on differential abundance of Tn5 insertions across input and output libraries using the Resampling method from Transit with -l, Loess correction, -PC 5, and -a for adaptive resampling (27). For Himar1 libraries both essential genes and differences between input and output libraries (conditionally essential) for data sets were determined using the Zero-Inflated Negative Binomial method (ZINB) from Transit (57). All data sets were normalized using the beta geometric distribution (-norm betageom). Genes in the ZINB output of Transit marked as “pan-essential” or “pan-growth defect” in the status columns were classified as essential. Genes that did not meet this criterion but had adjusted *P*-values < 0.05 and log2(fold change) < 0 were marked as conditionally-essential.

### Analysis of gene essentiality

Results from methods identifying essential and conditionally essential genes were processed in R v.4.1.1 and RStudio v.1.4.17. The FactorMineR v.2.8 package in R was used to conduct the quality assurance using multiple correspondence analysis (58). Essentiality calls were integrated using information from the pangenome and combined with S119 operon structures (36). To calculate essentiality scores for genes across lineages and conditions the essentiality calls from transposon sequencing results were used. The three groups, non-essential, conditionally essential, and essential, were seen as ordered (ordinal categorical data). Categories were assigned numeric values based on their order (non-essential = 0, conditionally essential = 1, essential = 2). Using the numeric values of essentiality, the lineage-weighted essentiality score was calculated in a two-step process. First, for each gene, the mean essentiality score within lineages (*emm*1, *emm*28, or *emm*49) was calculated. Secondly, the essentiality score across lineages was calculated as the mean of all lineages for the given gene. For *in vivo* and *in vitro* essentiality scores the mean for all data sets belonging to either one of the two categories was calculated for each gene, without correction for lineage. Having calculated an essentiality score, genes were defined as essential if an essentiality score ≥1.5. An essentiality score of <1.5 and >0.5 were considered conditionally essential (essential under some but not all conditions), and an essentiality score of ≤0.5 was considered non-essential. The cut-offs described were chosen relatively arbitrarily but were designed to give readers a logical boundary to grasp results, essentiality scores for genes are given in Table S2.

### Plotting, data wrangling, and statistical tests

Plots were produced using ggplot2 v.3.4.2, ggprism v.1.0.4, ggbreak v.0.1.2, networkD3 v.0.4, and htmlwidgets v.1.6.2 in R (59–63). Data handling was aided by the R package: reshape2 v.1.4.4 (64). Interactive html tables were produced using the DT v.0.28 package in R (65). Statistical tests were conducted in R. Wilcoxon test to compare medians of two samples were carried out using wilcox.test(), as a paired test (paired = T) if specified.

Resampling of observed data for the enrichment of essential genes across COG categories was done using the sample() and mclapply() functions in R, with no replacement in the sample, and seed: 34532 set in mclapply. Genes assigned to COG categories were counted once per assigned category. Linear regression for operon size and essentiality association was conducted using the lm() function in R, with operon essentiality as a function of operon size without fixing the intercept of the regression to the origin.

## Data Availability

Outputs from tools from Transit (ZINB, Resampling, Tn5Gaps) can be found under the DOI: doi.org/10.5281/zenodo.8401927. Data on the essentiality of genes and operons are provided as interactive html files as well as comma-separated tables. An additional table relating the pangenome cluster to locus_tags of reference genomes used is provided. Genes annotated with no gene name are in the pangenome given a name of group_####. Therefore, searching directly for a given gene name may not be possible.

## SUPPLEMENTAL MATERIAL

Data S1 spectrum.03240-23-s0001.csv Relation of annotations from genomes used for transposon sequencing.

Data S2 spectrum.03240-23-s0002.csv Table with essentiality of genes across strains.

Data S3 spectrum.03240-23-s0003.csv Difference in essentiality for 231 genes with large *in vivo* to *in vitro* essentiality scores.

Data S4 spectrum.03240-23-s0004.csv Table with overview of essentiality of operons.

Supplemental figures spectrum.03240-23-s0005.pdf Fig. S1 and S2.

Table S1 spectrum.03240-23-s0006.xlsx Summary statistics for transposons sequence libraries and list of RefSeq accession.

Table S2 spectrum.03240-23-s0007.xlsx Summary of how Clusters of Orthlogous Genes are summarised into larger groups.

HTML S1 spectrum.03240-23-s0008.html Interactive html table of genes.

HTML S2 spectrum.03240-23-s0009.html Interactive HTML of essentiality for genes.

HTML S3 spectrum.03240-23-s0010.html Interactive table with essentiality of operons.

ASM does not own the copyrights to Supplemental Material that may be linked to, or accessed through, an article. The authors have granted ASM a non-exclusive, world-wide license to publish the Supplemental Material files. Please contact the corresponding author directly for reuse.
