# Supplementary material for: Pangenome evaluation of gene essentiality in Streptococcus pyogenes
Source: Microbiol Spectr. 2024 Jul 16;12(8):e03240-23. doi: 10.1128/spectrum.03240-23 (PMC11323703; doi:10.1128/spectrum.03240-23)
Supplement: Supplemental figures — Fig. S1 and S2. [file spectrum.03240-23-s0005.pdf]

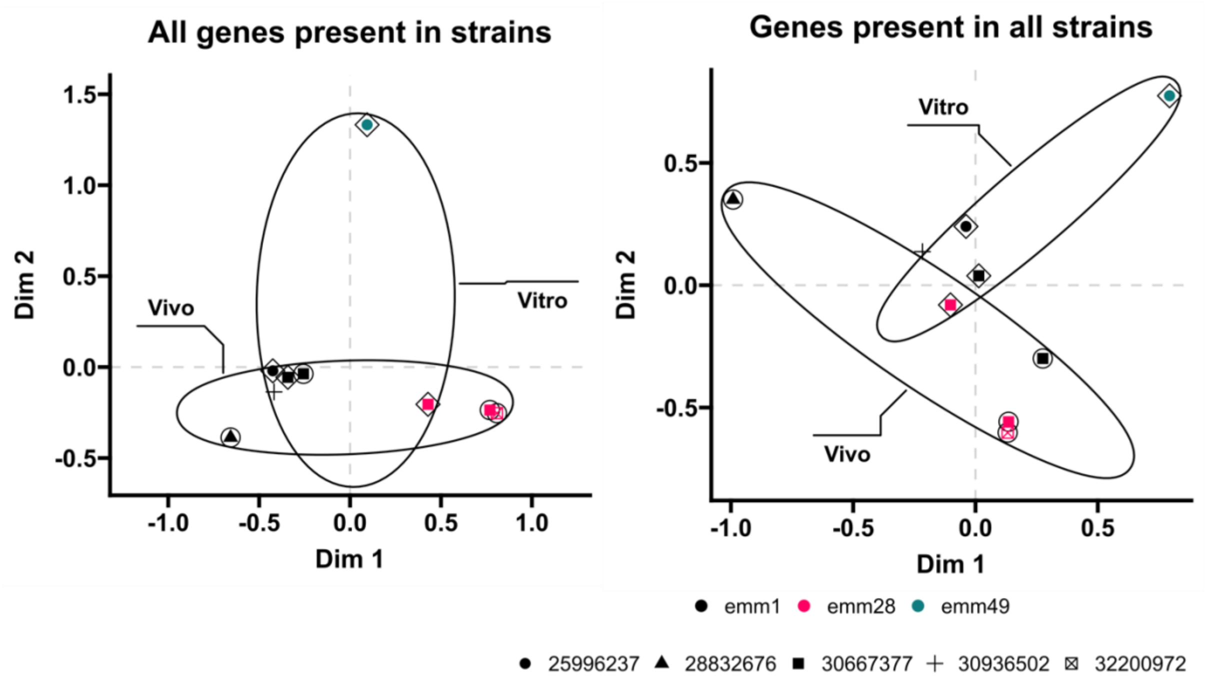

Supplementary figure 1 – Multiple correspondence analysis of transposon sequencing data. Each plot illustrates the first and second dimension returned from a multiple correspondence analysis of essentiality group for all genes present in strains (left) and genes present in all strains (right). Ellipses surround samples from *in vivo* and *in vitro* conditions. Point colours indicate *emm* lineage used in a dataset, and point shape indicate the PubMed ID from which data was obtained.

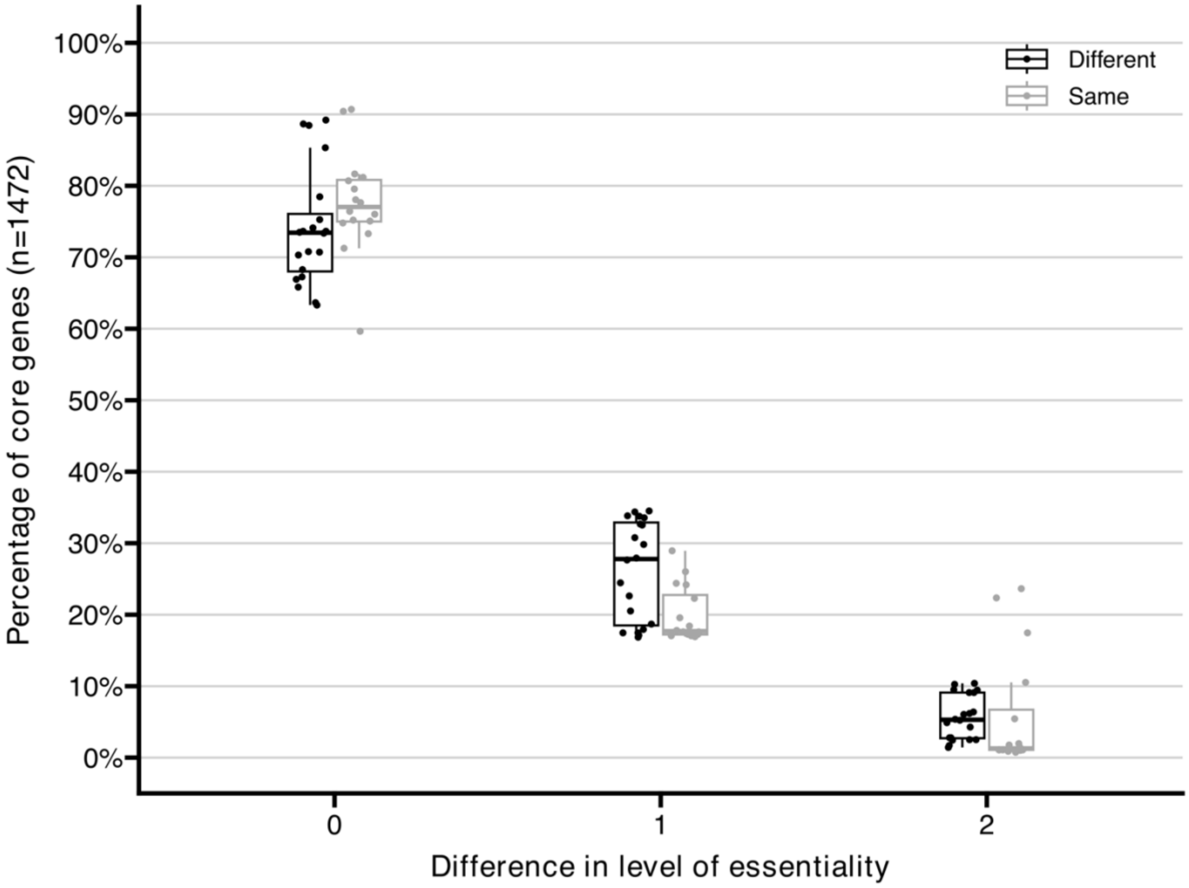

Supplementary figure 2 – Pairwise comparisons of essentiality calls from datasets of the same and different transposon type. Each dataset was compared to all other datasets. The horizontal axes indicate the difference in essentiality level across datasets (0: same essentiality level, 1: Conditionally-essential in one dataset, and essential or non-essential in the other. 2: non-essential in one dataset, and essential in the other). Each point indicates the percentage of core genes with a given difference in essentiality level that can be found for a pairwise comparison. Boxplots are given as summary for point placement. The legend and colours are given based on the transposon type of datasets compares. Black indicates datasets to have been produced using different transposon types and grey indicates a similar transposon type.
